# Editing the *kinesin-12* gene affects responses to Bt toxin Cry1Ac in *Helicoverpa zea*

**DOI:** 10.1038/s41598-025-29324-4

**Published:** 2025-11-26

**Authors:** Chan C. Heu, Kyle M. Benowitz, Luciano M. Matzkin, Carson W. Allan, Dannialle M. LeRoy, Xianchun Li, Bruce E. Tabashnik, Yves Carrière, Jeffrey A. Fabrick

**Affiliations:** 1 USDA ARS, U.S. Arid Land Agricultural Research Center, Maricopa, AZ 85138 USA; 2 College of Integrative Sciences and Arts, Arizona State University, Mesa, AZ 85212 USA; 3 Department of Entomology, University of Arizona, Tucson, AZ 85721 USA

**Keywords:** *Bacillus thuringiensis*, Transgenic Bt crops, Insecticide resistance, Corn earworm, Kinesin, CRISPR/Cas9 gene editing, Functional genomics, Agricultural genetics, Biotechnology, Molecular evolution, Mutation

## Abstract

Crops genetically engineered to produce insecticidal proteins from *Bacillus thuringiensis* (Bt) are used globally to manage key insect pests. However, the evolution of resistance to Bt proteins in at least 11 pest species has reduced the effectiveness of Bt crops. Resistance to crystalline (Cry) Bt proteins including Cry1Ac produced by Bt cotton is a major problem in *Helicoverpa zea* (also known as bollworm and corn earworm), one of the most economically damaging pests in the United States. A previous genome-wide association study identified a nonsense point mutation in a *kinesin-12* gene that was associated with resistance to Cry1Ac in a lab-selected strain of *H. zea*. Here, we used CRISPR/Cas9 gene editing to knock out the *kinesin-12* gene in a Cry1Ac-susceptible laboratory strain, which caused a 4.0-fold increase in resistance to Cry1Ac. Conversely, gene editing that repaired the natural *kinesin-12* nonsense mutation in a lab-selected resistant strain increased susceptibility to Cry1Ac by 3.8-fold. These complementary results provide compelling evidence that kinesin-12 plays a role in the mode of action of Cry1Ac against *H. zea*.

**Supplementary Information:**

The online version contains supplementary material available at 10.1038/s41598-025-29324-4.

## Introduction

Genetically engineered crops producing insecticidal proteins from *Bacillus thuringiensis* (Bt) are used globally to manage many important agricultural pests. Bt crops have improved pest management, increased yields, reduced management costs, and reduced the environmental impact of pest management by decreasing the use of conventional insecticide sprays^1–8^. Also, Bt cotton facilitated eradication of the invasive pest pink bollworm (*Pectinophora gossypiella*) from the commercial cotton-growing areas of the continental U.S. and northern Mexico^9^. However, the evolution of resistance by insect pests has reduced some of the benefits of Bt crops^10^. The number of cases of practical resistance to Bt crops is at least 31, involving 9 crystalline (Cry) Bt toxins and 11 pest species in 8 countries^10–13^.

Here we focus on the genetic basis of resistance to Bt toxin Cry1Ac in *Helicoverpa zea* (also known as bollworm and corn earworm), a lepidopteran that attacks many crops and is one of the most economically damaging pests in the United States^5,14,15^. Despite *H. zea* resistance to Bt cotton producing Cry1Ac being known as the first documented case of practical resistance to a Bt crop^10,16^ and extensive previous study, understanding of the genetic basis of this resistance has remained elusive. The most common and most potent mechanisms of resistance entail changes to larval midgut receptors (e.g., cadherin, aminopeptidase-N, alkaline phosphatase, and ABC transporters) that reduce their binding of Cry toxins^17,18^.

In *H. zea*, CRISPR/Cas9-mediated knockout of the ABC transporter ABCC2 caused up to 40-fold resistance to Cry1Ac^19^. Yet several genome-wide association studies (GWAS) found that mutations affecting ABCC2 and other candidate receptor genes were not major contributors to Cry1Ac resistance in field- or lab-selected *H. zea*^20–22^. Rather, studies of field-selected populations of *H. zea* found that in some but not all cases, Cry1Ac resistance is associated with amplification of a cluster of nine trypsin genes on chromosome 9^21,22^.

Studies of the lab-selected, highly resistant *H. zea* strain GA-R revealed that its resistance to Cry1Ac was associated with changes in some proteolytic digestive enzymes^23^ and naturally occurring mutations affecting a novel cadherin protein (CAD-86C)^24^ and a kinesin protein (kinesin-12)^20^. Benowitz et al.^20^ discovered that GA-R harbors a point mutation in *kinesin-12* that introduces a premature stop codon. They hypothesized that mutant *kinesin-12* was necessary but not sufficient for resistance to Cry1Ac. We are not aware of previous work with *H. zea* or any other insect directly testing the role of kinesin-12 in resistance or susceptibility to Bt toxins. Moreover, aside from the knockout of ABCC2 mentioned above^19^ and the knockout of ABCA2 causing resistance to Cry2Ab^25^, we are not aware of previous studies in which gene editing was used to test the role of any gene in resistance of *H. zea* to Bt toxins.

Here we used CRISPR/Cas9 gene editing to knock out *kinesin-12* in the susceptible LAB-S strain of *H. zea* to determine whether this would cause resistance to Cry1Ac. We also used gene editing to test the complementary hypothesis that insertion of the susceptible *kinesin-12* coding sequence from LAB-S would restore susceptibility in GA-R. We found that both types of gene editing caused minor yet statistically significant changes in responses to Cry1Ac in the predicted direction, which provides direct evidence that *kinesin-12* affects susceptibility to Cry1Ac in *H. zea*.

## Results

### Creation of the KinKO strain with *kinesin-12* knocked out in the susceptible LAB-S strain

**Fig. 1:**
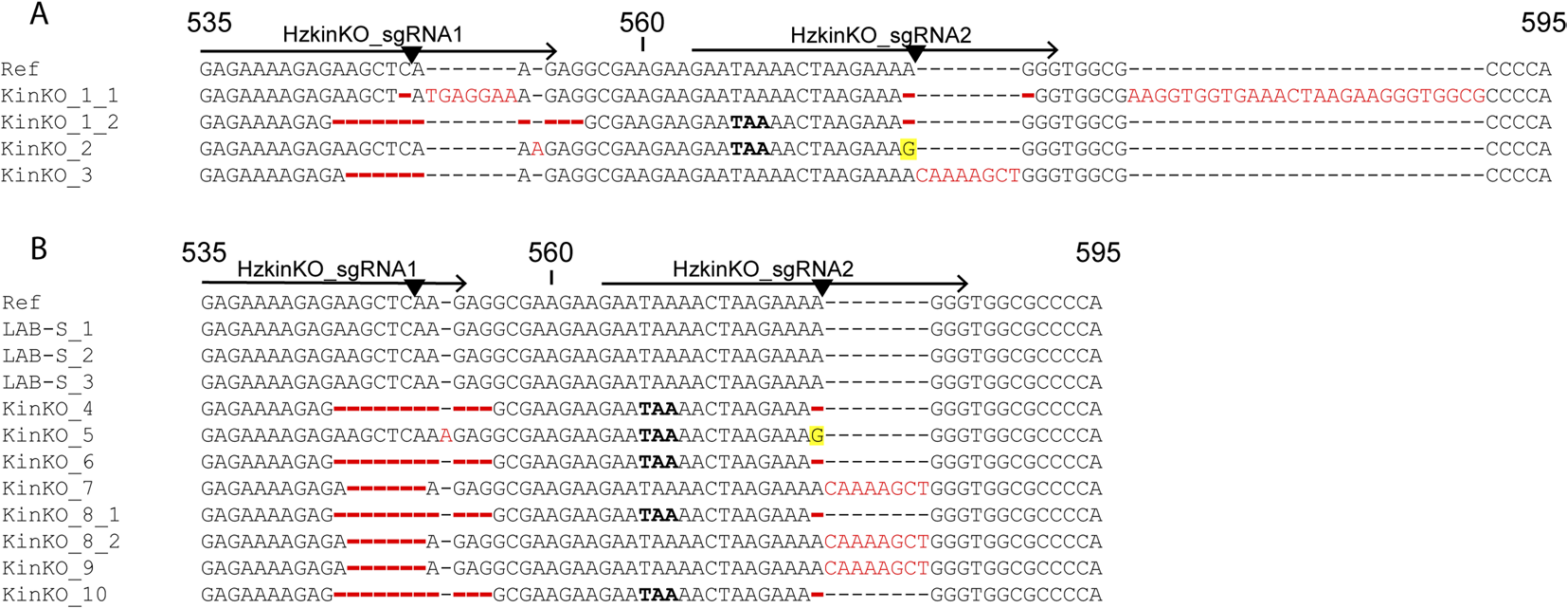
Mutations in *kinesin-12* in KinKO pre- and post- Cry1Ac diet bioassays. (**A**) Prior to Cry1Ac diet bioassays, three G_2_ KinKO larvae were selected for cloning and DNA Sanger sequencing of *kinesin-12* corresponding to the Cas9-sgRNA target sites. (**B**) Three LAB-S on untreated diet and seven KinKO individuals that survived (≥ 3rd instar) after 14 d on 3 or 10 µg Cry1Ac per cm^2^ diet were Sanger DNA sequenced. Numbers immediately following the sample names represent the individual and clone number (e.g. KinKO_8_2 is individual 8 clone 2). “Ref” is for reference sequence of *kinesin-12* (XM_047167698) from the *H. zea* strain Stark_Cry1AcR, which is identical to *kinesin-12* from LAB-S^21^. The arrows above the aligned nucleotide sequences correspond to the spacer sequence for the sgRNAs targeting *kinesin-12*, the black triangles indicate the predicted cut site, the bolded nucleotides (TAA) show the premature stop codons introduced by frameshift mutations, and the numbers indicate the nucleotide position of the coding sequence. Insertions are shown in red font, deletions are shown as red hyphens, and substitutions are highlighted in yellow.

We created the KinKO strain using CRISPR/Cas9 editing of the susceptible LAB-S strain of *H. zea* with two sgRNAs targeting *kinesin-12* simultaneously (Hz_kinKO_sgRNA1 and 2, Fig. 1A and Supplementary Table S1). Sanger sequencing of genomic DNA (gDNA) from four clones of three G_2_ KinKO larvae identified four alleles, each with a premature stop codon expected to yield a truncated, nonfunctional kinesin-12 protein (Fig. 1A). Sanger sequencing of gDNA from the three KinKO larvae showed no evidence of mutations in the three genomic regions outside of *kinesin-12* most likely to have off-target effects from the gene editing (Supplementary Fig. S1). Thus, it is unlikely that off-target mutations in KinKO relative to LAB-S were introduced by the gene editing.

###  Creation of the KinKI strain with a susceptible *kinesin-12* coding sequence inserted in the GA-R strain

**Fig. 2:**
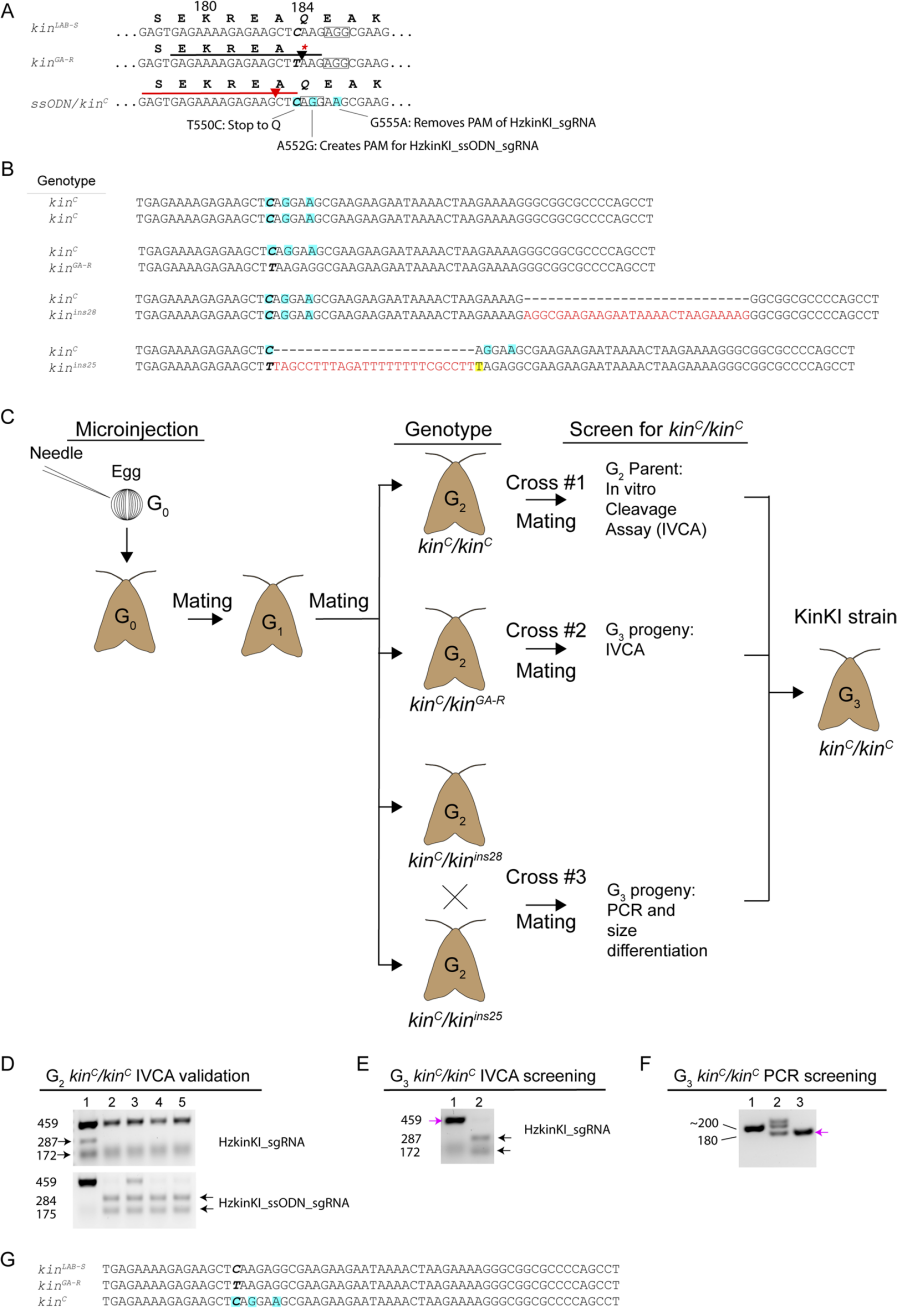
Generation and DNA genotyping of the KinKI strain. (**A**) Local sequence of the target region in *kin*^*LAB−S*^, *kin*^*GA−R*^, and ssODN/*kin*^*C*^. Black and red lines indicate the spacer sequence from HzkinKI_sgRNA or HzkinKI_ssODN_sgRNA, respectively, triangles indicate predicted cut sites and intended substitution mutations are highlighted in blue. (**B**) Four *kinesin-12* genotypes in G_2_ confirmed by Sanger DNA sequencing included: homozygous *kin*^*C*^/*kin*^*C*^, heterozygous *kin*^*C*^/*kin*^*GA−R*^, heterozygous *kin*^*C*^/*kin*^*ins28*^, and heterozygous *kin*^*C*^/*kin*^*ins25*^. (**C**) Crossing scheme used to generate the homozygous *kin*^*C*^/*kin*^*C*^ KinKI strain. (**D**) Cas9 in vitro cleavage assays used to validate G_2_ parental genotype for *kin*^*C*^/*kin*^*C*^. Lane 1 is GA-R, lanes 2–5 are the four G_2_ parents, arrows indicate diagnostic bands, and numbers show band size in bp. (**E**) Screening of the G_3_ progeny from the inbreeding of parents with *kin*^*C*^/*kin*^*GA−R*^. Representative image of a Cas9 in vitro cleavage assay using HzkinKI_sgRNA. Lanes 1 and 2 are G_3_ samples, pink arrow points to uncleaved template indicating *kin*^*C*^ homozygosity, black arrows indicate the two diagnostic bands for *kin*^*GA−R*^. (**F**) PCR screening of the G_3_ progeny *kin*^*C*^/*kin*^*C*^ from the cross between parents with *kin*^*C*^/*kin*^*ins28*^ and *kin*^*C*^/*kin*^*ins25*^. Each lane shows representative results of an individual G_3_ larva. A band at ~ 200 bp indicates *kin*^*ins25*^ or *kin*^*ins28*^, the 180 bp band indicates *kin*^*C*^, and the unexpected band at ~ 220 bp in Lane 2 likely indicates a nonspecific amplicon. Pink arrow identifies the single 180 bp band in lane 3 indicating the genotype *kin*^*C*^/*kin*^*C*^. Original images of unedited gels are presented in Supplementary Figure S3. (**G**) Representative *kinesin-12* sequences of larvae from LAB-S (*n* = 3) that survived on control diet, GA-R (*n* = 4), and G_3_ and G_4_ KinKI (*n* = 7) larvae that survived on 0.3, 30, or 300 µg Cry1Ac per cm^2^ diet in diet bioassays. For all aligned sequences, nucleotide position 550 is bolded and italicized, intended substitutions from the ssODN template are highlighted in blue, insertions have red font, and unintended substitution is highlighted in yellow.

 To create the KinKI strain, we used CRISPR/Cas9 editing with a sgRNA to eliminate the naturally occurring nonsense point mutation in the *kinesin-12* allele (*kin*^*GA−R*^) in the resistant GA-R strain (Fig. 2A, Supplementary Fig. S2). We first used HzkinKI_sgRNA to introduce a double-strand break in *kinesin-12* (Supplementary Table S1) of GA-R and initiate homology directed repair (HDR) by providing the HzkinKI_ssODN as a template to repair *kinesin-12* (Fig. 2A, Supplementary Table S1). We used this process to insert a synthetic sequence of *kinesin-12* in GA-R that encodes the wild-type kinesin-12 protein from the LAB-S strain. The synthetic sequence contains wild-type sequence from LAB-S to replace the mutation in GA-R (C550T, previously reported incorrectly as C546T^20^ and two novel synonymous single base pair substitutions (A552G and G555A) that enabled us to distinguish between this “corrected” allele (*kin*^*C*^) and the wild-type allele from LAB-S. In addition, the mutation A552G introduces a new PAM sequence to facilitate screening of the HzkinKI_ssODN using an in vitro cleavage assay with sgRNA HzkinKI_ssODN_sgRNA. Sanger sequencing of gDNA from KinKI G_2_ larvae (four clones per individual from 150 individuals) revealed four *kinesin-12* genotypes: (1) *kin*^*C*^/*kin*^*C*^ homozygotes and heterozygotes with *kin*^*C*^ and (2) *kin*^*GA−R*^, (3) *kin*^*ins28*^ with a 28-bp insertion, or (4) *kin*^*ins25*^ with a 25-bp insertion (Fig. 2B).

We used 150 *kin*^*C*^/*kin*^*C*^ G_3_ moths (63 males and 87 females) from three G_2_ crosses to create the KinKI strain (Fig. 2C). In cross #1, *kin*^*C*^/*kin*^*C*^ G_2_ moths (two males and two females) were allowed to mate. After oviposition, the *kin*^*C*^/*kin*^*C*^ genotype of the four G_2_ parents was confirmed by in vitro Cas9 cleavage (Fig. 2D, upper panel, lanes 2–5, and Supplementary Fig. S3A). A total of 34 *kin*^*C*^/*kin*^*C*^ G_3_ moths (15 males and 19 females) from cross #1 were pooled and included to start the KinKI strain.

In cross #2, G_2_ moths genotyped as *kin*^*C*^*/kin*^*GA−R*^ (4 males and 4 females) were allowed to mate and produce G_3_ offspring (Fig. 2C, cross #2). We nondestructively screened 96 G_3_ larvae from cross #2 using the Cas9 in vitro cleavage assay with the HzkinKI_sgRNA and excluded all larvae harboring at least one *kin*^*GA−R*^ allele (Fig. 2E, lane 2, and Supplementary Fig. S3B). In total, 33 G_3_ moths (13 males and 20 females) from cross #2 were pooled and added to the KinKI strain.

In cross #3, G_2_ moths genotyped nondestructively as either *kin*^*C*^*/kin*^*ins28*^ (10 males and 5 females) or *kin*^*C*^*/kin*^*ins25*^ (2 females) were pooled and allowed to mate. We then screened 396 G_3_ larvae using nondestructive PCR followed by agarose gel electrophoresis to distinguish among the genotypes (Fig. 2F, and Supplementary Fig. S3C). Individuals with alleles *kin*^*ins25*^ or *kin*^*ins28*^ were excluded. From cross #3, 83 G_3_ individuals (35 males and 48 females) genotyped as *kin*^*C*^/*kin*^*C*^ were added to the KinKI strain. Sanger sequencing of the full-length *kinesin-12* gDNA from three KinKI larvae revealed no off-target site mutations (Supplementary Fig. S1).

### Resistance to Cry1Ac in KinKO relative to its susceptible parent strain LAB-S

**Fig. 3:**
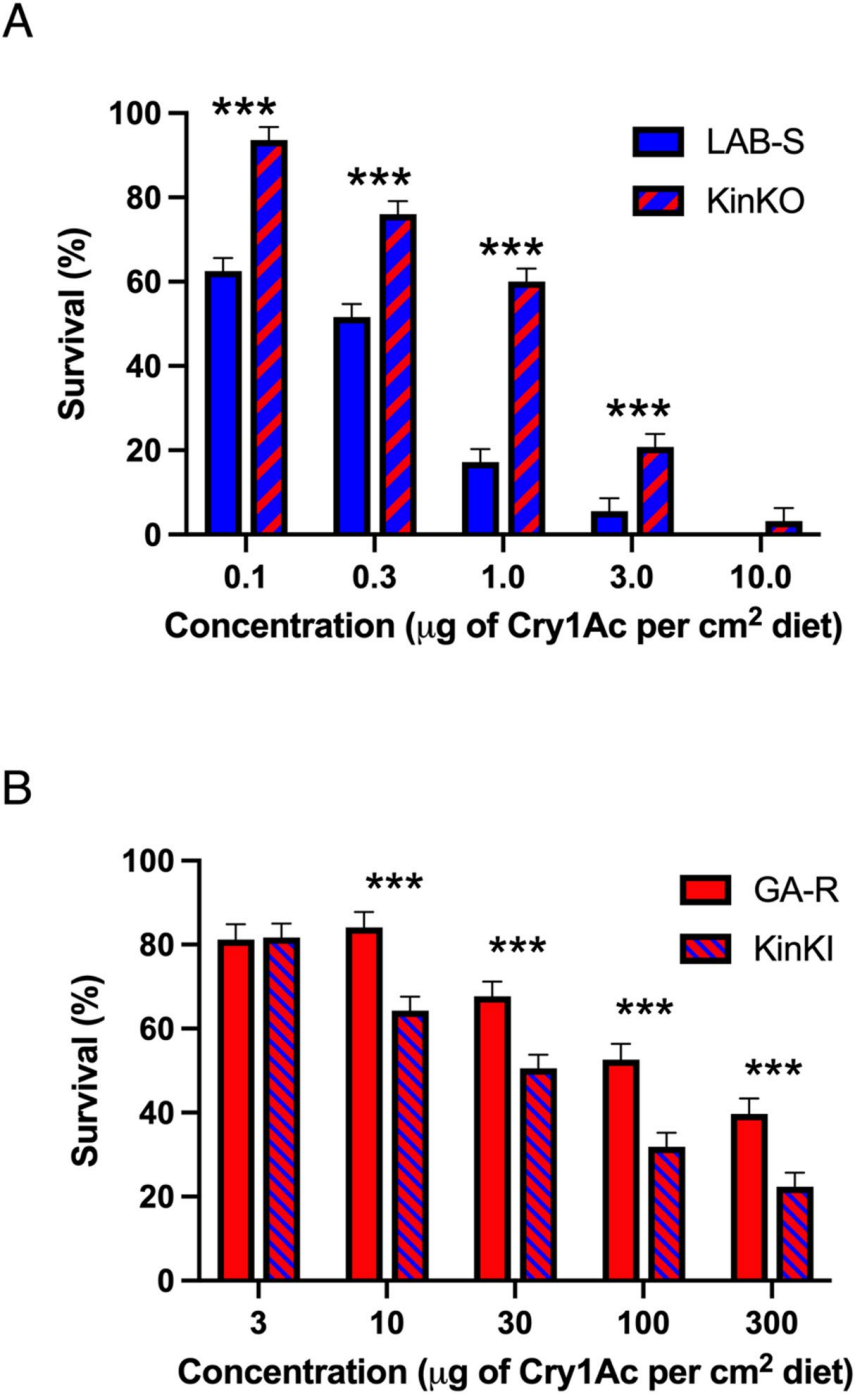
Effects of editing *H. zea kinesin-12* on responses to Cry1Ac. (**A**) Pairwise comparison of LAB-S and KinKO survival percentages for criterion ≥ 3rd instar using least squares means contrasts. A total of 128 larvae were test for each concentration in each of two replicates. (**B**) Pairwise comparisons of GA-R and KinKI survival percentage using criterion ≥ 3rd instar using least squares means contrasts for pooled replicates. We tested 128 neonates per strain at each concentration for each of three replicates, with the following exceptions: 112 larvae for LAB-S and GA-R at 3 µg Cry1Ac per cm^2^ diet and KinKI at 0 µg Cry1Ac per cm^2^ diet and *n* = 96 for GA-R at 100 µg Cry1Ac per cm^2^ diet for replicate 1. In replicate 3 for GA-R, 64 larvae were tested for 3, 10, 100, and 300 µg Cry1Ac per cm^2^ diet and 96 larvae for 30 µg Cry1Ac per cm^2^ diet. *** means the *P* value is < 0.001.

The concentration of Cry1Ac killing 50% of larvae (LC_50_, in µg Cry1Ac per cm^2^ diet with 95% fiducial limits [FL]) was significantly greater for KinKO (0.89, 0.71–1.11) than LAB-S (0.22, 0.13–0.30), yielding a resistance ratio (RR) of 4.0 for KinKO relative to LAB-S (Table 1). These results support the hypothesis that knockout of *kinesin-12* in LAB-S caused resistance to Cry1Ac. In addition, survival was significantly higher for KinKO than LAB-S at four of the five concentrations of Cry1Ac analyzed separately (Fig. 3A). For example, survival at 1 µg Cry1Ac per cm^2^ diet was 60% for KinKO versus 17% for LAB S (*P* < 0.0001, Fig. 3A). The only exception occurred at the highest concentration tested (10 µg Cry1Ac per cm^2^ diet) at which survival was 3.2% for KinKO versus 0% for LAB-S (*P* = 0.082, Fig. 3A, Supplementary Table S2). The RR was more than 100-fold lower for KinKO (4.0) than resistant strain GA-R (418, Table 1), which indicates that knockout of *kinesin-12* was not sufficient to achieve the level of resistance in GA-R.

**Table 1:** Responses of four strains of *H. zea* to Cry1Ac in diet bioassays.

Strain^a^	*n* ^b^	Slope (SE)^c^	LC_50_ (95% FL)^d^	RR^e^
LAB-S	2992	1.9 (0.1)	0.22 (0.13–0.30)	1.0
KinKO	1216	1.6 (0.1)	0.89 (0.71–1.11)	4.0
KinKI	3024	0.8 (0.3)	24.3 (18.5–32.5)	111
GA-R	2320	0.5 (0.0)	91.9 (45.6–259)	418

^a^LAB-S, susceptible strain; KinKO, *kinesin-12* knockout strain; KinKI, *kinesin-12* knock-in strain in which the mutation in GA-R was repaired; GA-R, resistant strain.

^b^Number of larvae tested.

^c^Slope of the concentration-mortality line and its standard error (SE).

^d^Concentration killing 50% of larvae (LC_50_) and 95% fiducial limits (FL) in µg Cry1Ac per cm^2^ diet.

^e^Resistance ratio, the LC_50_ of each strain divided by the LC_50_ of LAB-S.

### Susceptibility to Cry1Ac greater for KinKI than its resistant parent strain GA-R

The LC_50_ of Cry1Ac (in µg Cry1Ac per cm^2^ diet with 95% FL) was 3.8-fold lower for KinKI (24.3, 18.5–32.5) than GA-R (91.9, 45.6–259) (Table 1), consistent with the hypothesis that repairing the *kinesin-12* mutation in GA-R increased susceptibility to Cry1Ac. In addition, survival was significantly lower for KinKI than GA-R at four of the five concentrations tested (10, 30, 100 and 300 µg Cry1Ac per cm^2^ diet, Fig. 3B). For example, survival at 100 µg Cry1Ac per cm^2^ diet was 32% for KinKI versus 53% for GA-R (*P* < 0.0001, Fig. 3B). The only concentration at which no significant difference occurred between strains was the lowest concentration tested (3 µg Cry1Ac per cm^2^ diet) at which survival was 81% for GA-R and 82% for KinKI (Fig. 3B, Supplementary Table S3). The RR relative to LAB-S is 111 for KinKI versus 418 for GA-R (Table 1), which indicates that repairing the *kinesin-12* mutation in GA-R did not fully restore susceptibility to Cry1Ac.

### Genotypes of survivors from bioassays

Sanger sequencing of 24 bioassay survivors confirmed the expected genotypes in each of the four strains studied. All six larvae from LAB-S that survived on diet treated with 0.3–3 µg Cry1Ac per cm^2^ diet had only the wild-type *kin*^*LAB−S*^ allele. Each of the seven larvae from KinKO that survived on diet treated with 10 µg Cry1Ac per cm^2^ diet had mutations affecting both sgRNA target sites (Fig. 1B) that correspond with mutations found in the three G_2_ larvae genotyped before the bioassay (Fig. 1A). For survivors on diet treated with 300 µg Cry1Ac per cm^2^ diet, the GA-R larvae had only the *kin*^*GA−R*^ allele (*n* = 4) and the KinKI larvae had only the *kin*^*C*^ allele (*n* = 7).

## Discussion

This study provides direct evidence that the *kinesin-12* gene, which was identified via GWAS as a candidate for contributing to Cry1Ac resistance^20^, affects Cry1Ac intoxication in *H. zea.* CRISPR/Cas9 knockout of *kinesin-12* in the susceptible LAB-S strain caused resistance to Cry1Ac while repairing the natural mutation in this gene in the resistant strain GA-R increased susceptibility to Cry1Ac.

Benowitz et al.^20^ discovered that the point mutation introducing a premature stop codon in *kinesin-12* occurred in all genomic sequencing reads from 30 GA-R larvae and in none of the reads from 30 LAB-S larvae. They also found that in the heterogeneous GA-RS strain created by crossing GA-R and LAB-S, at least one copy from GA-R of both genetic markers closest to *kinesin-12* occurred in all larvae that were deemed resistant based on the criterion of surviving to third or a subsequent instar after 7 d on diet containing 10 µg Cry1Ac per cm^2^ diet. By contrast, these two markers were in Hardy-Weinberg equilibrium in the GA-RS larvae that were deemed susceptible based on the criterion that they were first instars after 7 d on diet containing 1 µg Cry1Ac per cm^2^ diet. Thus, approximately 75% of the susceptible GA-RS larvae had one or two copies of the mutant *kinesin-12* allele from GA-R. These results spurred the suggestion that mutant *kinesin-12* is necessary but not sufficient for resistance to Cry1Ac, with survival to third instar on diet containing 10 µg Cry1Ac per cm^2^ diet as the criterion for resistance^20^. The results here support the hypothesis that mutant *kinesin-12* is not sufficient for resistance based on that criterion, because survival on diet with 10 µg Cry1Ac per cm^2^ diet did not differ significantly between KinKO (3.2%) and LAB-S (0%).

By contrast, the results with KinKI do not support the hypothesis that mutant *kinesin-12* is necessary for resistance. Survival on diet containing 10 µg Cry1Ac per cm^2^ diet was 0% for LAB-S versus 64.3% for KinKI, which had the wild-type coding sequence for kinesin-12. Also, KinKI had an RR of 111 relative to LAB-S. The results of Benowitz et al.^20^ that generated this hypothesis are based on the heterogeneous GA-RS strain. During the rearing of GA-RS for many generations without exposure to Cry1Ac, resistance to Cry1Ac decreased but the frequency of the *kinesin-12* mutation did not, which implies that GA-RS lost one or more mutations at loci other than *kinesin-12* that contribute to resistance in GA-R^20^. Thus, in the genetic background of GA-RS, *kinesin-12* was necessary for resistance. In comparison, KinKI is expected to have retained all of the resistance-conferring mutations from GA-R except for the *kinesin-12* mutation. The results with KinKI show that in this genetic background, the *kinesin-12* mutation was not necessary for resistance.

Unlike the >100-fold resistance often associated with disruption of Bt toxin midgut receptors such as cadherin and ABC transporters^17^, knockout of *kinesin-12* caused only 4-fold resistance to Cry1Ac in the KinKO strain of *H. zea*. One hypothesis to explain this result is that functional redundancy occurs such that other kinesins or other proteins may partially complement a nonfunctional kinesin-12, which reduces the level of resistance caused by knocking out this protein. For example, ABCC2 and ABCC3 are functionally redundant in *Helicoverpa armigera* and *Ostrinia furnacalis*, such that knocking out either gene alone caused little or no resistance to Cry1Ab or Cry1Ac, respectively, but knocking out both genes caused >1,000-fold resistance^26,27^. Also, because of functional redundancy in the toxic pathways of Cry1Ab against *O. furnacalis*, knocking out cadherin alone did not cause resistance while knocking out cadherin and ABCC2 caused an RR of 9300^27^.

The 111-fold resistance to Cry1Ac in KinKI, compared to 418-fold in GA-R, supports the hypothesis that resistance to Cry1Ac in GA-R involves mutations in one or more loci other than *kinesin-12*^20^. Fritz et al.^24^ identified nonsynonymous substitutions in a cadherin gene (*cad-86C*) in GA-R, but mutations in this gene and 10 other genes previously implicated in lepidopteran resistance to Cry toxins were not associated with resistance to Cry1Ac in GA-R in the GWAS conducted by Benowitz et al.^20^. In an earlier study, trypsin activity and Cry1Ac activation by midgut proteases was lower in GA-R than LAB-S^23^, suggesting that insufficient toxin activation might be a resistance mechanism in GA-R. A cluster of trypsin genes on chromosome 9 that was amplified in some populations of *H. zea* with field-selected resistance to Cry1Ac^21,22^ was not amplified in GA-R^21^. Whereas this study provides strong evidence for a minor role of mutant *kinesin-12* in resistance to Cry1Ac in GA-R, mutations in this gene were not associated with field-selected resistance to Cry1Ac^20,21^. Thus, the results here confirm and extend the previous conclusion of a mismatch in the genetic basis of field-selected versus lab-selected resistance to Cry1Ac in *H. zea*^20^.

Kinesins can function in maintaining cell structure, intracellular transport, and other cell functions^28–32^. Interactions between kinesin-12 and myosin-II, a non-muscle actin-associated motor protein, are important for many cellular processes^30,31,33^. Myosin-II cross-links the actin filamentous matrix that supports the parallel actin bundles critical for maintaining microvilli structures of mouse small intestinal tissue^34^. If something similar occurs in *H. zea* larvae, knockout of kinesin-12 might disrupt microvilli in the larval midgut and reduce binding of Cry1Ac to receptors. Alternatively, given that kinesin-12 functions in intracellular transport in human hepatocytes^32^, knockout of *H. zea* kinesin-12 might reduce binding of Cry1Ac by interfering with transport of receptor proteins to the surface of larval midgut cells. In *Helicoverpa armigera* and *P. gossypiella*, mislocalization of cadherin midgut receptors caused by cellular mistrafficking contributes to Cry toxin resistance^35–38^. To better understand the normal role of kinesin-12 in susceptible insects and how disruption of this protein contributes to Bt resistance, further studies are needed including comparison of binding of Cry1Ac in insects with and without functional kinesin-12.

## Materials and methods

### Insects

 Two previously described strains^39,40^ of *H. zea* were used for gene editing: a susceptible laboratory strain (LAB-S) from Benzon Research (Carlisle, PA), and a Cry1Ac-resistant strain (GA-R) that was founded with 180 larvae collected on Cry1Ab corn in Georgia in 2008, selected in the lab with Cry1Ac^39^, and harbors the C550T nonsense mutation in the *kinesin-12* gene^20^. We name the allele containing this mutation *kin*^*GA−R*^. Strains were reared either on Southland diet (Southland Products, Inc., Lake Village, AR) or modified wheat germ-based diet^41^ at 28 °C with 40–60% humidity and 14 h light:10 h dark. Moths were provided water containing 10% sucrose and cheese cloth sheets for oviposition.

### Cry1Ac

 The source of Cry1Ac was MVPII (Dow Agrosciences, San Diego, CA), a liquid formulation of protoxin encapsulated in recombinant *Pseudomonas fluorescens*^42^. Because the entire active toxin of MVPII is identical to that of the Cry1Ac holotype^40^, we refer to MVPII as Cry1Ac.

### Design and synthesis of single guide RNAs (sgRNAs) for editing *kinesin-12*

 Using previously described strategies^25,43^, we designed four sgRNAs targeting *kinesin-12* (Supplementary Table S1). We used HzkinKO_sgRNA1 and HzkinKO_sgRNA2 to knock out *kinesin-12* in LAB-S (Fig. 1). To repair the mutation in *kinesin-12* of GA-R^20^, we used HzkinKI_sgRNA to introduce a double-strand break (DSB) between nucleotide position 550 and 551 (Fig. 2A), which led to homology directed repair (HDR). After HDR, HzkinKI_ssODN_sgRNA targeted the repaired *kinesin-12* (Fig. 2A) to facilitate screening for HDR.

All sgRNA DNA templates were synthesized as gBlock DNA (Integrated DNA Technologies, Coralville, Iowa) and contained a T7 RNA polymerase binding site, a 19–20 bp gene-specific target sequence, and the 80-bp common stem-loop tracrRNA sequence. The gBlock DNA templates were used to synthesize sgRNA using the HiScribe T7 High Yield RNA Synthesis Kit (New England Biolabs, Ipswich, MA). Transcribed sgRNAs were treated with DNase I for 20 min at 37 °C and purified using RNAClean XP (Thermo Fisher Scientific) following the manufacturer’s protocol.

### Design of the donor template single-stranded deoxynucleotide (ssODN)

 An ssODN (HzkinKI_ssODN) was designed according to the recommended guidelines from Bollen et al.^44^. It corresponded to a sense strand (i.e., 5’ to 3’ in the direction of the open reading frame), was 100 nucleotides in length, had asymmetrical homology arms (i.e., 65 nucleotides at the 5’ end and 35 nucleotides at the 3’ end), and both 5’ and 3’ ends were chemically modified using Alt-R HDR (Integrated DNA Technology, Coralville, Iowa) to reduce enzymatic degradation (Supplementary Fig. S1). HzkinKI_ssODN served as the template for HDR to revert the nonsense mutation C550T, found in GA-R (i.e., *kin*^*GA−R*^), to T550C (i.e., *kin*^*LAB−S*^), thus changing the TAA stop codon at amino acid position 184 to the CAG codon for glutamine (Fig. 2A). In addition, HzkinKI_ssODN included two synonymous mutations relative to *kin*^*LAB−S*^ including: (1) A552G which introduced a new protospacer adjacent motif (PAM) site to enable efficient detection of the HDR-mediated repair; and (2) G555A which eliminated the PAM site recognized by HzkinKI_sgRNA (Fig. 2A). All three substitutions resulted in a genetically engineered *kinesin-12* gene with a reduced chance of HzkinKI_sgRNA-directed Cas9 retargeting.

### Embryo microinjection

Either one or both sgRNAs were singly mixed with the Alt-R *Streptococcus pyogenes* HiFi Cas9 nuclease V3 (Integrated DNA Technologies) (50–100 ng/µL sgRNA to 100–200 ng/µL Cas9) and incubated at room temperature for 15 min to allow the formation of the Cas9-ribonucleoprotein (RNP) complex. When two sgRNAs were used, mixing occurred following formation of each individual RNP complex. The RNP complexes were held on ice and immediately used for injections.

As previously described^25^, *H. zea* embryos were collected for 45 min by placing microscope glass coverslips (24 × 40 mm, Corning Inc., Corning, NY) directly on top of screened containers harboring 5 male and 5 female adults. Because adult *H. zea* females oviposit and affix eggs to the coverslips, no additional manipulation of the embryos was needed prior to injection. An IM-300 microinjector (Narishige International USA, Amityville, NY) equipped with an Olympus IMT-2 inverted microscope (Olympus Corporation, Center Valley, PA) was used to inject newly laid eggs (less than 1 h old) with approximately 100–200 picoliters of RNP solution. Quartz needles (Sutter Instrument Co, Novato, CA) were backfilled with 3 µL of the RNP mix using Eppendorf Microloader pipette tips and the fine points of the capillary needles were opened by gently grazing the needle against the side of the coverslip. Post injection, coverslips containing injected embryos were held in 100 × 15 mm Petri dishes containing 1% agarose at 28 °C until G_0_ neonates emerged approximately 3 d after.

### Generating the *Helicoverpa zea kinesin-12* knockout strain KinKO

 LAB-S eggs (*n* = 291 total) were microinjected with a solution containing Cas9 (200 ng/µL) complexed with both HzkinKO_sgRNA1 (100 ng/µL) and HzkinKO_sgRNA2 (100 ng/µL). Newly emerged G_0_ neonates were transferred to individual 30 mL translucent polystyrene cups containing 10 mL of Southland diet and held at 28 °C (14:10 L: D). Once G_0_ larvae reached 4th or 5th instar, we used in vitro T7 endonuclease assays to screen for individuals harboring mutations within the corresponding sgRNA target region of the *kinesin-12* gene. We used microscissors to make a superficial nick of a proleg from 150 individual G_0_ larvae, from which we collected 1 µL of hemolymph into dilution buffer and DNA Release Agent (20:0.5 vol: vol) from the Thermo Scientific™ Phire Tissue Direct PCR Master Mix kit (Thermo Fisher Scientific). We PCR amplified the *kinesin-12* region of interest using gene specific primers (Supplementary Table S1) and thermal cycling: one cycle of 98 °C for 5 min; 40 cycles of 98 °C for 5 s, 55 °C for 5 s, 72 °C for 10 s, and a final extension of 72 °C for 1 min. To test for potential Cas9-induced mutations, each PCR amplicon was denatured (95 °C for 5 min), slowly annealed (from 95 to 85 °C at a ramp rate of -2 °C/s followed by 85 to 25 °C at a ramp rate of -0.1 °C/s), and incubated with T7 endonuclease I (New England Biolabs) at 37 °C for 15 min. Reactions were separated by electrophoresis on 1% agarose gels and imaged using an Azure c600 gel imager (Azure Biosystems, Dublin, CA). Because the T7 endonuclease only cleaves reannealed amplicons harboring mismatches (e.g., indels, substitutions), which are indicative that non-homologous end joining (NHEJ) has occurred, DNA band profiling using agarose gel electrophoresis allowed for detection of potential knockout mutations in the *kinesin-12* gene. In total, we obtained 120 larvae showing diagnostic bands, which were allowed to pupate. These were placed into six rearing cages, each containing 10 female and 10 male pupae. Adults arising from these pupae were provided 10% sugar and cheese cloth on which to oviposit G_1_ eggs.

A second round of screening for knockout of *kinesin-12* gene was performed on G_1_ larvae using Sanger DNA sequencing. G_1_ neonates were reared on Southland diet and 4th or 5th instar larvae were sampled by collecting hemolymph, as outlined above. From 150 G_1_ larvae, we conducted PCR of the *kinesin-12* target region of by HzkinKO_sgRNA1 and 2 and purified the amplicons for direct Sanger DNA sequencing (Retrogen, San Diego, CA). In total, sixty G_1_ larvae (30 males and 30 females), each having two *kinesin-12* knockout alleles (i.e., *kin*^*KO*^ alleles), were crossed to establish the KinKO strain.

Potential off-target Cas9 cut sites from HzkinKO_sgRNA1 and HzkinKO_sgRNA2 were predicted based on CRISPOR^45^ using the *H. zea* Stark_Cry1AcR (GCF_022581195.2) and the GA-R (GCA_022343045.1) genome assemblies. Tentative off-target sequences were chosen if they (1) contained a PAM site, and (2) had three or less base pair mismatches to the seed region (N_1_-N_10_) of HzkinKO_sgRNA1 and HzkinKO_sgRNA2, respectively. Then, three G_1_ larvae were selected and screened for potential off-target mutations using Phire (Thermo Fisher Scientific) PCR and gene-specific primers (Supplementary Table S1). PCR amplicons were subcloned into the pJET1.2/blunt vector (ClonJET PCR Cloning Kit, Thermo Fisher Scientific), transformed into One Shot TOP10 Chemically Competent *E. coli* (Thermo Fisher Scientific), and grown on LB agar plates with 100 ng/µL of carbenicillin. Four plasmids per amplicon were purified using QIAprep Spin MiniPrep kit (Qiagen, Hilden, Germany) and Sanger DNA sequenced.

### Generating the *Helicoverpa zea kinesin-12* knock-in strain (KinKI)

 We injected 45 G_0_ eggs from resistant strain GA-R that were less than 1 h old with ~ 50–100 pL of a cocktail mixture containing 200 ng Cas9, 100 ng HzkinKI_sgRNA, and 100 ng HzkinKI_ssODN per µL. Neonates were reared on untreated modified wheat germ diet^41^ and moths were pooled for mating and oviposition. Fourth instar G_2_ larvae (*n* = 150) were used in the first round of genotyping, with hemolymph used as the templates for PCR to amplify the *kinesin-12* target region for subcloning into pJET1.2/blunt vector, as described above. Plasmid DNA was purified and four clones per individual were Sanger DNA sequenced to identify the genotype.

Screening for larvae homozygous for the repaired *kin*^*C*^ allele was performed using either in vitro Cas9 cleavage or PCR genotyping depending on the genotype (Fig. 2). For both verification methods, gDNA templates corresponding to *kinesin-12* target regions were amplified with gene specific primers using Phire PCR with the same thermal cycling as indicated above. For the in vitro Cas9 cleavage assays, 10 µL of each PCR reaction was mixed with 2 µL of 10x NEB 3.1 buffer [1x], 1 µL of 300 ng/µL Cas9 [15 ng/µL], 1 µL of 200 ng/µL of sgRNA [10 ng/µL], and brought up to a total volume of 20 µL per sample with nuclease-free water. Samples were incubated at 37 °C for 15 min and separated by agarose gel electrophoresis. Either HzkinKI_sgRNA or HzkinKI_ssODN_sgRNA were the sgRNA added to the mix to identify the *kin*^*GA−R*^ and *kin*^*C*^ allele, respectively. For individuals verified to harbor *kinesin-12* DNA insertions, PCR and agarose gel electrophoresis were used to discriminate differences in the size of amplicons between the repaired *kin*^*C*^ and insertion (*kin*^*ins*^) mutant genotypes. Only G_3_ adults homozygous for *kin*^*C*^ were used to propagate the KinKI strain.

### Diet overlay bioassays

 We tested one neonate per well in bioassay trays (BIO-BA-128, Pitman, NJ) on Southland diet to which we added 40 µl of 0.1% Triton X-100 per well. Trays were covered with Pull N’ Peel covers (BIO-CU-16, Pitman, NJ) and held at 28 °C (14 h light:10 h dark). After 7 d, living larvae of third or subsequent instars were considered survivors whereas dead larvae and live first or second instars were considered dead. We used this survival criterion to facilitate comparison with bioassay results from Benowitz et al.^20^.

We compared KinKO (G_3_) versus LAB-S using six concentrations: 0 (control), 0.1, 0.3, 1, 3, or 10 µg Cry1Ac per cm^2^ diet (*n* = 128 larvae for each concentration in each of two replicates conducted seven weeks apart; total *n* = 1728 larvae).

We compared KinKI (G_4_, G_5_, and G_6_) versus GA-R (corresponding generations F_130_, F_131_, and F_132_) using three replicates of bioassays conducted seven weeks apart. As an internal control, LAB-S was also tested simultaneously in each of the three replicates. The concentrations of Cry1Ac used in the bioassays for KinKI, GA-R, and LAB-S included 0, 0.3, 1, 3, 10, 30, 100, and 300 µg Cry1Ac per cm^2^ diet. We tested KinKI and GA-R on diet with 500 µg Cry1Ac per cm^2^ diet only in replicate 2. We tested 128 neonates per strain at each concentration for each replicate with the following exceptions: For replicate 1, *n* = 112 for LAB-S and GA-R at 3 µg Cry1Ac per cm^2^ diet and KinKI at 0 µg Cry1Ac per cm^2^ diet; and *n* = 96 for GA-R at 100 µg Cry1Ac per cm^2^ diet. In replicate 3 for GA-R, *n* = 64 for 3, 10, 100, and 300 µg Cry1Ac per cm^2^ diet and 96 for 30 µg Cry1Ac per cm^2^ diet.

### Genotypes of larvae from bioassays

Larvae surviving in diet bioassays after 14 d (LAB-S on 3 µg Cry1Ac per cm^2^ diet and KinKO on 10 µg Cry1Ac per cm^2^ diet) and after 7 d (LAB-S on 0.3 µg Cry1Ac per cm^2^ diet, GA-R and KinKI on 300 µg Cry1Ac per cm^2^ diet) were Sanger DNA sequenced to determine their *kinesin-12* genotype.

### Statistical analyses of bioassay data

 Survival was adjusted for control mortality using Abbott’s correction^46^. The concentration of Cry1Ac killing 50% of larvae (LC_50_) and its 95% fiducial limits (FLs) were determined using probit analysis in PoloSuite version 2.1^47^. Although the survival criterion used here for calculating LC_50_ value (i.e., number of live larvae that were third or subsequent instars) differs from the criterion used by Welch et al.^40^ (i.e., number of larvae that were second or subsequent instars), these two criteria yielded similar resistance ratios. Two LC_50_ values were considered significantly different based on the conservative criterion of no overlap between their FLs. The resistance ratio was calculated by dividing the LC_50_ for a strain by the LC_50_ for LAB-S. For each of the two pairwise comparisons between strains (LAB-S vs. KinKO and GA-R vs. KinKI), we used least squares means contrasts from ANOVA (see Supplementary methods) conducted using JMP Pro (2021) to assess whether adjusted survival (%) differed significantly between strains at each Cry1Ac concentration.

## Supplementary Information

Below is the link to the electronic supplementary material.

Supplementary Material 1

## Data Availability

The full-length *H. zea kinesin-12* cDNA sequence (PV485217.1) corresponding to the *kin*^C^ allele generated during the current study is available in the NCBI GenBank repository (https://www.ncbi.nlm.nih.gov/genbank/). Sequence data supporting the findings of this study are available from the corresponding author upon request.
