# Supplementary material for: Editing the kinesin-12 gene affects responses to Bt toxin Cry1Ac in Helicoverpa zea
Source: Sci Rep. 2025 Nov 26;15:45378. doi: 10.1038/s41598-025-29324-4 (PMC12748845; doi:10.1038/s41598-025-29324-4)
Supplement: Supplementary file 1 — Supplementary Material 1 [file 41598_2025_29324_MOESM1_ESM.docx]

**SUPPLEMENTARY INFORMATION**

**Editing the *kinesin-12* gene affects responses to Bt toxin Cry1Ac in *Helicoverpa zea***

Chan C. Heu^1^, Kyle M. Benowitz^2^, Luciano M. Matzkin^3^, Carson W. Allan^3^, Dannialle M. LeRoy^1^, Xianchun Li^3^, Bruce E. Tabashnik^3^, Yves Carrière^3^, Jeffrey A. Fabrick^1,*^

^1^ USDA ARS, U.S. Arid Land Agricultural Research Center, Maricopa AZ, USA 85138

^2^ College of Integrative Sciences and Arts, Arizona State University, Mesa, AZ USA 85212

^3^ Department of Entomology, University of Arizona, Tucson, AZ USA 85721

^*^ Corresponding author:

Jeffrey A. Fabrick

USDA ARS, U.S. Arid Land Agricultural Research Center

21881 N. Cardon Lane

Maricopa, AZ 85138, USA

Phone: 520-316-6335

Email: [jeff.fabrick@usda.gov](mailto:jeff.fabrick@usda.gov)

**SUPPLEMENTARY INFORMATION includes:**

**Supplementary Tables S1-S3**

**Supplementary Figures S1-S3**

**Statistical Methods for Tables S2 and S3**

**Reference cited in SUPPLEMENTARY INFORMATION**

**Supplementary Table S1. Oligonucleotide primer, guide, and single-stranded oligonucleotides (ssODN) DNA sequences.**

| **Oligo name** | **Oligo Sequence (5' to 3')** | **Purpose** |
| --- | --- | --- |
| 3HzKin-5 | ACCGAACTTGAGGCAAGAAA | PCR amplification of target region for cloning/sequencing and for generating target for in vitro cleavage assay |
| 6HzKin-3 | GGAGGACCGCTGCTTTTACT |  |
| HzKin12_FL_F1 | GTGGTGTGCAACGTTATACTA | PCR amplification of full-length kinesin-12 |
| HzKin12_FL_R1 | CGCTCAACCATTATAAACTGATA |  |
| HzKinKI_diff_F1 | GACAAACAACGAGCTGCAGA | PCR genotyping of KinKI progeny from parental crosses |
| HzKinKI_diff_R1 | AGCAGTTGCTTGAGCCAAAG |  |
| HzKin_4_19_OT1_fwd1 | AGTGTTTGAGATCACTGCCA | Detection of HzkinKO_sgRNA1 off target site in the intergenic region (chromosome 8, 11965282-11965300)^a^ |
| HzKin_4_19_OT1_rev1 | CCCCATCTTGCAGAAACAAGT |  |
| HzKin_4_19_OT3_fwd1 | AGCTCTGTTTCTACTCGAGT | Detection of HzkinKO_sgRNA1 off target site in LOC124644465^a^ |
| HzKin_4_19_OT3_rev1 | AAATCTGAGGGTGACAAACAC |  |
| HzKin_6_OT1_fwd1 | GTGCTAAGAGTTTGCGATCC | Detection of HzkinKO_sgRNA2 off target site LOC124639406^a^ |
| HzKin_6_OT1_rev1 | TGGAACAAACACTGCCATTGC |  |
| HzKin_6_OT2_fwd1 | ATTTGAAGTGGCACTGCTTC | Detection of HzkinKO_sgRNA2 off target site in the intergenic region (chromosome 2, 89446-89465)^a^ |
| HzKin_6_OT2_rev1 | GGCGACTCTCGAATACCTTTA |  |
| HzKin_6_OT3_fwd1 | CGCAAGGACAAGAAGTAACG | Detection of HzkinKO_sgRNA2 off target site in a lncRNA (ENSH2EG000050679.1)^a^ |
| HzKin_6_OT3_rev1 | ACGAACTTCCGAAAGCTTTAA |  |
| HzkinKO_sgRNA1^b^ | GAGAAAAGAGAAGCTCAAG | Induce NHEJ in kin^LAB-S^ |
| HzkinKO_sgRNA2^b^ | GAATAAAACTAAGAAAAGGG | Induce NHEJ in kin^LAB-S^ |
| HzkinKI_sgRNA^b^ | GAGAAAAGAGAAGCTTAAG | Induce double strand break in kin^GAR^ |
| HzkinKI_ssODN_sgRNA ^b^ | GAGTGAGAAAAGAGAAGCTC | In vitro cleavage assay to confirm kin^C^ genotype |
| HzkinKI_ssODN | ACTAGCGCAAAAAGAACAAGCTAAACAGGAGAAGTTGCGAAAAGAGAGTGAGAAAAGAGAAGCTCAGGAAGCGAAGAAGAATAAAACTAAGAAAAGGGTG^c^ | ssODN template used to repair kin^GAR^ |

^a^ Sequence obtained from GCF_022581195.2.

^b^ Only target-specific spacer sequence is shown.

^c^ ssODN was modified at the 5' and 3' end with Alt-R by Integrated DNA Technology.

**Supplementary Table S2. Effects of Cry1Ac on survival of *Helicoverpa zea* from the LAB-S and KinKO strains in diet overlay bioassays.**

| **Concentration (μg Cry1Ac per cm^2^ diet)** | **% Survival (SE)^a,b^** | | **P^c^** |
| --- | --- | --- | --- |
|  | LAB-S | KinKO |  |
| 0.1 | 62.5 (3.1) | 93.6 (3.1) | <0.0001 |
| 0.3 | 51.6 (3.1) | 76.0 (3.1) | <0.0001 |
| 1 | 17.2 (3.1) | 60.0 (3.1) | <0.0001 |
| 3 | 5.5 (3.1) | 20.8 (3.1) | 0.0009 |
| 10 | 0 | 3.2 (3.1) | 0.47 |

^a^ Percentages were transformed using Abbott’s method (Tabashnik et al.)^1^. Standard errors (SE) are shown in parentheses.

^b^ After 7 d, living larvae of third or subsequent instars were considered survivors whereas dead larvae and live first or second instars were considered dead.

^c^ Probability value from contrast between LAB-S and KinKO for each concentration.

**Supplementary Table S3. Effects of Cry1Ac on survival of *Helicoverpa zea* from the GA-R and KinKI strains in diet overlay bioassays.**

|  | **% Survival (SE)^a,b^** | |  |
| --- | --- | --- | --- |
| **Concentration (μg Cry1Ac per cm^2^ diet)** | **GA-R** | **KinKI** | **P^c^** |
| 3 | 81.2 (3.7) | 81.7 (3.3) | 0.92 |
| 10 | 84.1 (3.7) | 64.3 (3.3) | <0.0001 |
| 30 | 67.7 (3.5) | 50.5 (3.3) | 0.0004 |
| 100 | 52.6 (3.8) | 31.9 (3.3) | <0.0001 |
| 300 | 39.7 (3.7) | 22.4 (3.3) | 0.0006 |

^a^ Percentages were transformed using Abbott’s method (Tabashnik et al.)^1^. Standard errors (SE) are shown in parentheses.

^b^ After 7 d, living larvae of third or subsequent instars were considered survivors whereas dead larvae and live first or second instars were considered dead.

^c^ Probability value from contrast between LAB-S and KinKO for each concentration.

**Supplementary Figure S1. Off-target screening of KinKO.** Alignments and chromatograms of actual Sanger DNA sequencing results with the *kinesin-12* spacer sgRNA sequences. A) Two potential off-targets of HzkinKO_sgRNA1 within intergenic regions (chromosome 8; position 11965282-11965300 and LOC124644465). B) Three potential off-targets of HzkinKO_sgRNA2 were found in LOC124539406, an intergenic region (chromosome 2, position 89446-89465), and within a long noncoding RNA (lncRNA) (ENSH2EG000050679.1). Arrows indicate direction of the guide in relation to the orientation of the reference genome (GCF_022581195.2), highlighted bases indicate mismatched base-pairing within the spacer sequences, and a red box identified the sequences of potential off-target sites within the chromatograms.

**
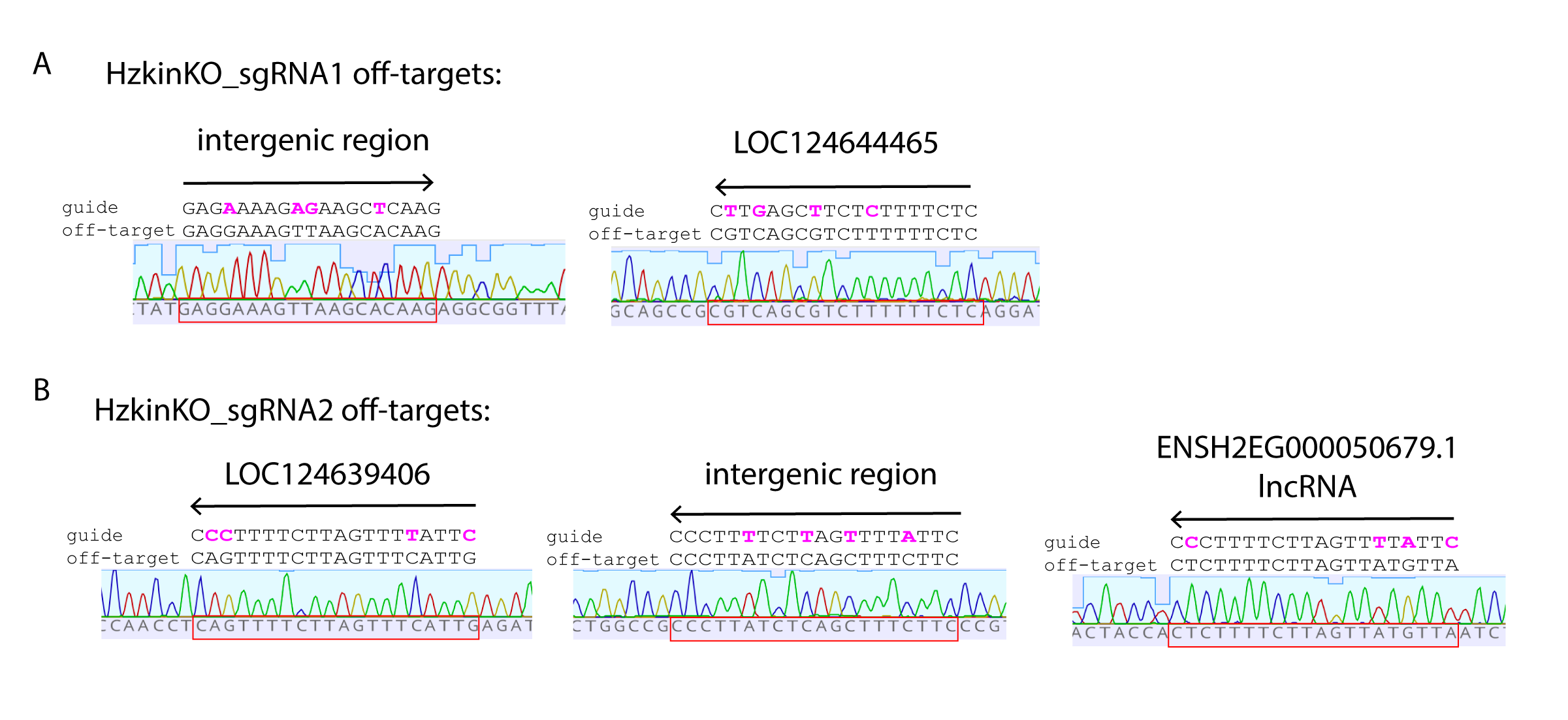
**

**Supplementary Figure S2. Full-length DNA sequence of *kin^C^*.** Chromatogram of *kin^C^* aligned with *kin^GA-R^*. Arrow heads indicate the three introduced “corrective” mutations in *kin^C^*, green bars indicate the positions of exons 1 and 2, and the yellow bar indicates the position of a single intron.


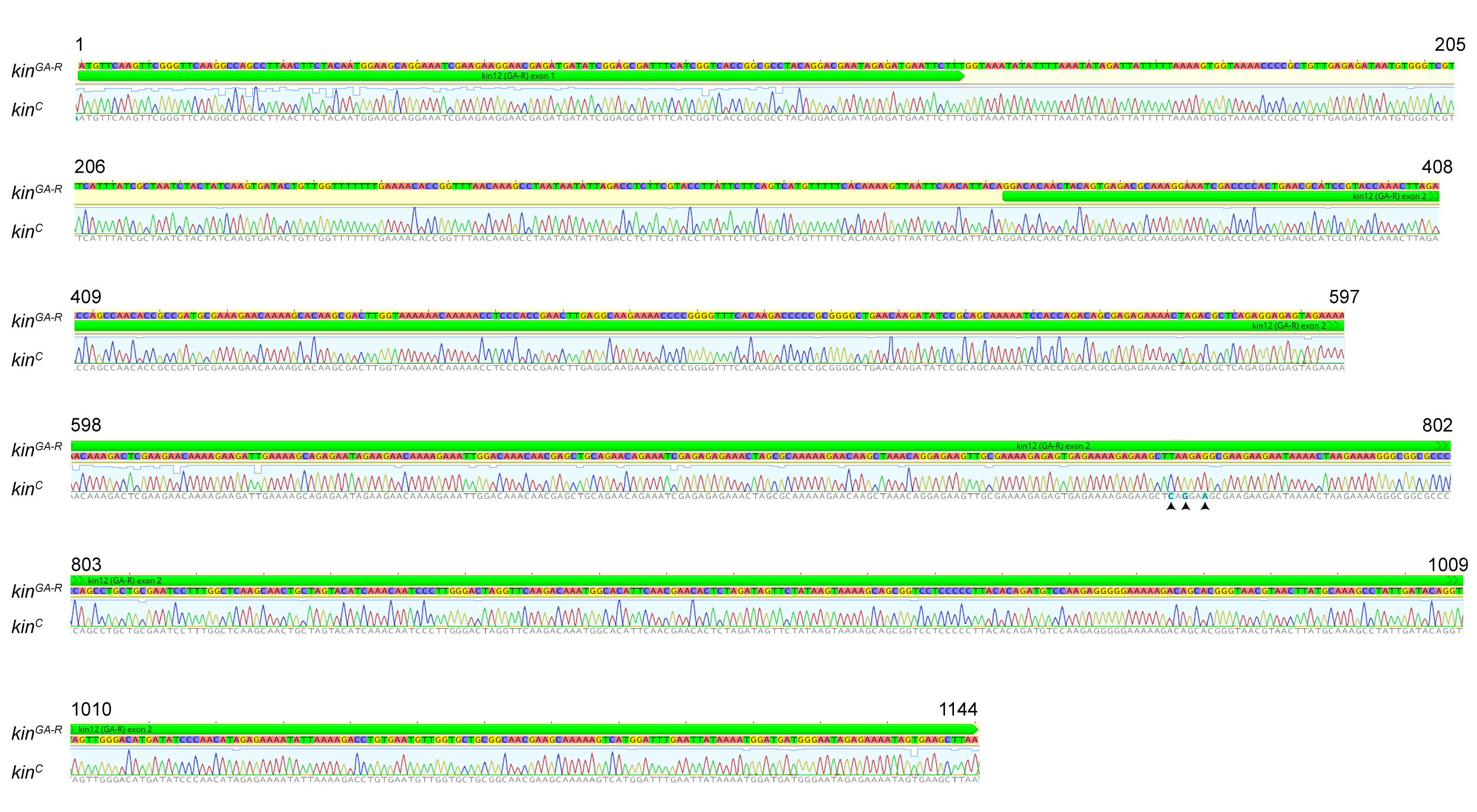


**Supplementary Figure S3. Unaltered images of agarose gel electrophoresis of KinKI genotyping.** A) Raw image of an in vitro cleavage assay of G_2_ parental samples using HzkinKI_sgRNA and HzkinKI_ssODN_sgRNA. Box 1 and 2 indicates top and bottom panels, respectively, in Fig. 2D. B) Raw image of an in vitro cleavage assay of G_3_ progeny from parental cross of *kin^C^/kin^GA-R^* genotype. Box indicates the cropped portion used as a representative image in Fig. 2E. C) A representative image of the PCR amplification and size differences on an agarose gel used to differentiate homozygous (*kin^ins25^* or *kin^ins28^*), heterozygous (*kin^C^/kin^ins25^* or *kin^ins28^*), or homozygous (*kin^C^*). Box indicates the cropped portion used in Fig. 2F.


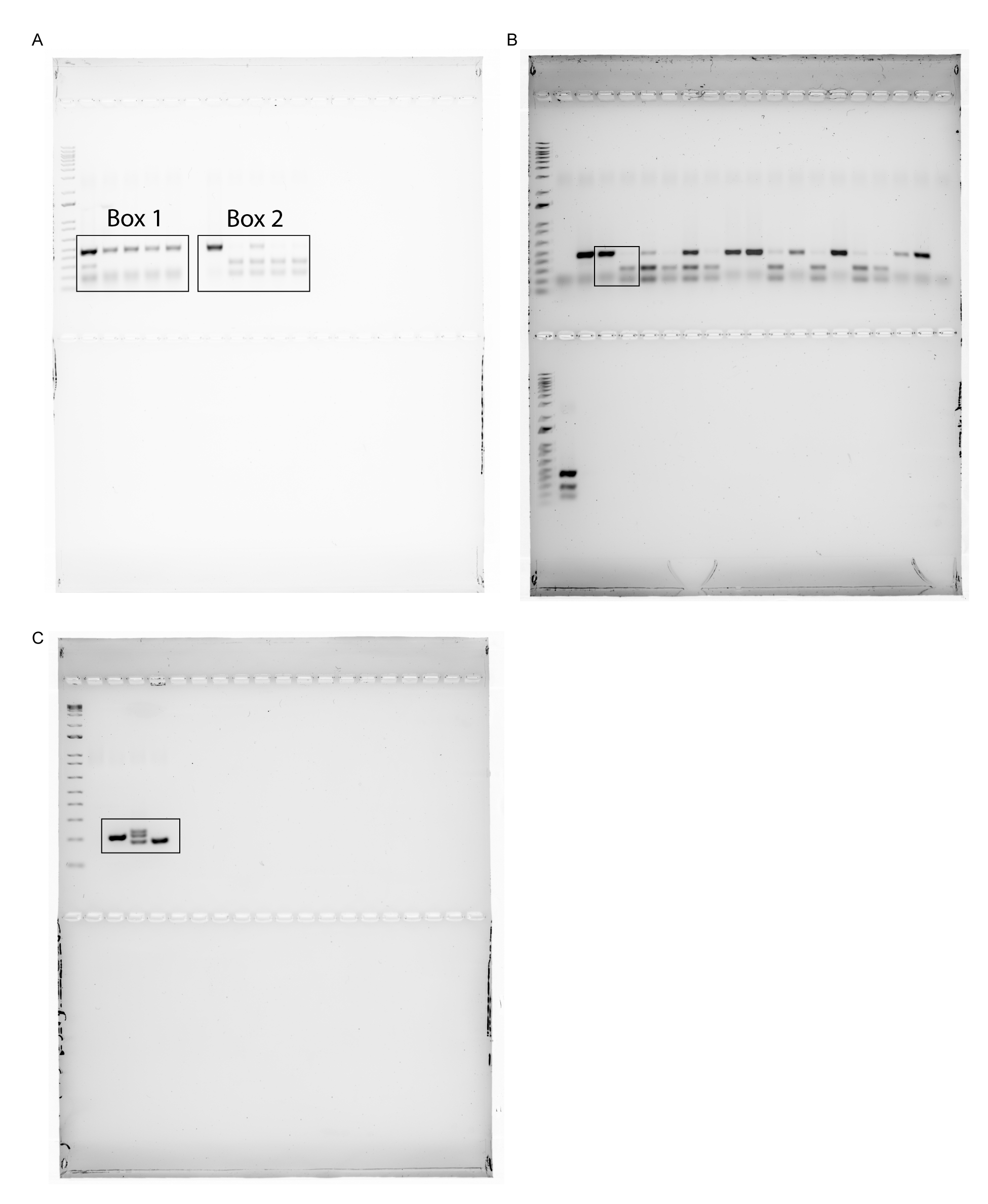


**Methods for statistical analyses**

**Table S2.** To compare responses to Cry1Ac between LAB-S and KinKO, we used two-way ANOVA to evaluate factors influencing the adjusted percentage survival. Explanatory variables were insect strain, Cry1Ac concentration, and their interaction. Least squares means contrasts were used to compare the adjusted percentage of survival between strains at each Cry1Ac concentration.

**Table S3.** To compare responses to Cry1Ac between GA-R and KinKI we used three-way ANOVA to analyze the pooled data for the three trials (each conducted on a different date). We only considered Cry1Ac concentrations evaluated in all three trials. Explanatory variables were trial, insect strain, Cry1Ac concentration, and the interaction between insect strain and Cry1Ac concentration. Least squares means contrasts were used to compare the adjusted percentage survival between strains at each Cry1Ac concentration.

**Reference**

1. Tabashnik, B. E. et al. Frequency of resistance to *Bacillus thuringiensis* in field populations of pink bollworm. *Proc. Natl. Acad. Sci. USA* **97**, 12980-12984; 10.1073/pnas.97.24.12980 (2000).
